# Importance of *Campylobacter jejuni* FliS and FliW in Flagella Biogenesis and Flagellin Secretion

**DOI:** 10.3389/fmicb.2017.01060

**Published:** 2017-06-12

**Authors:** Katarzyna A. Radomska, Marc M. S. M. Wösten, Soledad R. Ordoñez, Jaap A. Wagenaar, Jos P. M. van Putten

**Affiliations:** ^1^Department of Infectious Diseases and Immunology, Utrecht UniversityUtrecht, Netherlands; ^2^Wageningen Bioveterinary ResearchLelystad, Netherlands; ^3^WHO Collaborating Centre for Campylobacter/OIE Reference Laboratory for CampylobacteriosisUtrecht, Netherlands

**Keywords:** *Campylobacter jejuni*, flagellar motility, flagellar chaperone, flagellin, FliS, FliW

## Abstract

Flagella-driven motility enables bacteria to reach their favorable niche within the host. The human foodborne pathogen *Campylobacter jejuni* produces two heavily glycosylated structural flagellins (FlaA and FlaB) that form the flagellar filament. It also encodes the non-structural FlaC flagellin which is secreted through the flagellum and has been implicated in host cell invasion. The mechanisms that regulate *C. jejuni* flagellin biogenesis and guide the proteins to the export apparatus are different from those in most other enteropathogens and are not fully understood. This work demonstrates the importance of the putative flagellar protein FliS in *C. jejuni* flagella assembly. A constructed *fliS* knockout strain was non-motile, displayed reduced levels of FlaA/B and FlaC flagellin, and carried severely truncated flagella. Pull-down and Far Western blot assays showed direct interaction of FliS with all three *C. jejuni* flagellins (FlaA, FlaB, and FlaC). This is in contrast to, the sensor and regulator of intracellular flagellin levels, FliW, which bound to FlaA and FlaB but not to FlaC. The FliS protein but not FliW preferred binding to glycosylated *C. jejuni* flagellins rather than to their non-glycosylated recombinant counterparts. Mapping of the binding region of FliS and FliW using a set of flagellin fragments showed that the C-terminal subdomain of the flagellin was required for FliS binding, whereas the N-terminal subdomain was essential for FliW binding. The separate binding subdomains required for FliS and FliW, the different substrate specificity, and the differential preference for binding of glycosylated flagellins ensure optimal processing and assembly of the *C. jejuni* flagellins.

## Introduction

Flagella-driven motility is an important virulence trait of many bacterial pathogens and is required to establish infections (Josenhans and Suerbaum, 2002). The flagella of different bacterial species exhibit diverse characteristics but share a common basic architecture. Flagella can be divided into three parts: the basal body complex, the hook, and the flagellar filament (Bardy et al., 2003; Chevance and Hughes, 2008). The basal body anchors the flagellum in the cell envelope and contains the flagellar export machinery and the rotary motor that together with the set of stators generates the torque (Che et al., 2014). The flagellar hook forms a curved protein filament that under the right angle connects the basal body with the flagellar filament, enabling efficient rotation of the flagellum. The mature filament that protrudes from the bacterial surface may consist of as many as 20,000 copies of one or more flagellin subunit proteins (Fernández and Berenguer, 2000; Evans et al., 2014). Structural analysis of bacterial flagellins indicates that the proteins are folded into distinct morphological domains, D0–D3 (Vonderviszt et al., 1991; Samatey et al., 2001). The N- and C-terminal D0 and D1 domains of flagellins of different bacterial species share many features. The D0 and D1 domains are crucial for flagellin polymerization and are mainly buried within the structure of the intact flagellum (Samatey et al., 2001). In many species, the D1 domain contains the region that is recognized by innate immune Toll-like receptor 5 (TLR5) (Yoon et al., 2012). The D2 and D3 domains are more variable among bacterial species and are largely surface exposed (Malapaka et al., 2007). In a number of bacterial species, these domains are decorated with sugar moieties that provide further microheterogenity between strains (Thibault et al., 2001; Power and Jennings, 2003).

The major bacterial foodborne pathogen *Campylobacter jejuni* carries one flagellum at each pole (Pead, 1979) The flagellum of *C. jejuni* is comprised of seven protofilaments consisting of FlaA and FlaB subunits, whereas the flagella in enteropathogens form a helix of 11 protofilaments (Galkin et al., 2008). The flagellin of *C. jejuni* is heavily glycosylated (Nuijten et al., 1991; Ewing et al., 2009) and lacks the TLR5 binding site (Andersen-Nissen et al., 2005; de Zoete et al., 2010). The flagellum can serve as an export machinery to secrete proteins into the environment (Christensen et al., 2009; Neal-McKinney et al., 2010). One of the secreted proteins is the FlaC flagelin, which carries D0–D1 flagellin domains but largely lacks the variable D2–D3 domains. FlaC has been implicated in the *C. jejuni* invasion of host cells (Song et al., 2004). Why FlaC is secreted rather than assembled into the growing flagellum is unknown, but indicates different intracellular processing of the distinct types of flagellin.

One of the major bacterial challenges during the formation of flagella is the coordination of the complex flagella assembly process. This requires timely transcription of subsets of flagellar genes and fine-tuning of synthesis, intracellular processing, folding and export of flagellar proteins, and the formation of a functional flagella fiber (Aldridge and Hughes, 2002). One group of proteins that is critical in flagella biogenesis are the flagellar chaperones. These proteins interact with distinct flagellar components including the hook-filament junction proteins FlgK/FlgL, the filament-cap protein FliD, and the flagellin subunits (Fraser et al., 1999; Auvray et al., 2001; Khanra et al., 2016). The flagellar chaperones are central in preventing aggregation of flagellar proteins, including their targeting to the export gate of flagellar type 3 secretion system (T3SS) (Bange et al., 2010; Kinoshita et al., 2013). The paradigm of flagellar chaperones is the flagellin-specific FliS protein. FliS binds to the C-terminal domain of flagellins of amongst others *Salmonella enterica* (Ozin et al., 2003; Muskotál et al., 2006) and *Aquifex aeolicus* (Evdokimov et al., 2003) and is required for flagella assembly and bacterial motility. Protein crystal structures of *A. aeolicus, Helicobacter pylori*, and *Bacillus subtilis* indicate that the structure of FliS proteins is highly helical (Evdokimov et al., 2003; Badger et al., 2005; Lam et al., 2010). Despite the conserved nature of the FliS chaperone, differences exist between FliS of different species. FliS from *A. aeolicus* acts as a monomer (Evdokimov et al., 2003), whereas FliS from *S. enterica* forms a homodimer (Auvray et al., 2001). In *H. pylori* FliS forms a complex with the co-chaperone molecule HP1076 which may stimulate the folding activity of FliS (Lam et al., 2010). The flagellar chaperones of different species may also display clear substrate specificity (Fraser et al., 1999; Auvray et al., 2001). In *S. enterica* and *Yersinia pseudotuberculosis* the FliS chaperone interacts with the anti-sigma factor FlgM that modulates the transcription of the late (sigma^28^-dependent) flagellar genes (Galeva et al., 2014; Xu et al., 2014). *Vibrio parahaemolyticus* even expresses two different FliS proteins that preferentially interact with flagellins that form polar and lateral flagella, respectively (McCarter, 2004).

Another intracellular flagellar protein that interacts with bacterial flagellins is the FliW protein (Titz et al., 2006; Mukherjee et al., 2011). In *C. jejuni* and *B. subtilis*, this protein binds to flagellin as part of a feedback loop that controls the biosynthesis of flagellins at the post-transcriptional level. FliW adjusts the intracellular flagellin levels through a reciprocal interaction with the flagellin and the post-transcriptional regulator CsrA. In *C. jejuni* and *B. subtilis* CsrA interacts with 5′-UTR of flagellin transcripts and inhibits the translation initiation. Binding of FliW to CsrA resolves this inhibition (Mukherjee et al., 2011; Dugar et al., 2016; Radomska et al., 2016). In *B. subtilis* the interaction of FliS with the flagellin indirectly antagonizes CsrA activity by enhancing flagellin secretion. The increased flagellin export reduces the level of intracellular flagellin and this promotes the FliW partner switch (Mukherjee et al., 2013). In *C. jejuni* CsrA activity seems to be regulated by FliW-CsrA-*flaA* mRNA cross-talk, whereby the abundance of flagellin transcript was demonstrated to titrate CsrA activity (Dugar et al., 2016).

Currently, FliW is the only known regulator of CsrA activity in *C. jejuni* and *B. subtilis*. In the majority of bacterial species, CsrA is controlled by a set of two small non-coding RNAs, *csrB* and *csrC*, which in turn are regulated by the BarA/UvrY two-component system. The *C. jejuni* genome lacks both the antagonizing small RNAs and a two-component system of the similar function (Kulkarni et al., 2006; Romeo et al., 2013). In contrast to the non-coding RNAs, which compete with flagellin transcript for CsrA binding, FliW antagonizes the CsrA activity in a non-competitive manner (Altegoer et al., 2016; Mukherjee et al., 2016).

*Campylobacter jejuni* also contains a FliS ortholog that is important for bacterial motility (Golden and Acheson, 2002) and interacts with the flagellins of *C. jejuni* in a yeast two-hybrid screen (Parrish et al., 2007). The FliS and FliW proteins of *B. subtilis* can interact simultaneously with the Hag flagellin (Mukherjee et al., 2013). In this species, both proteins appear to interact with the carboxyterminal end of the flagellin (Evdokimov et al., 2003; Titz et al., 2006). It is unknown whether *C. jejuni* FliS and FliW have the same substrate specificity, and bind to the same or different regions of the flagellins. The aim of the present study was to better understand the intracellular processing of *C. jejuni* flagellins by defining the binding properties and substrate specificities of the *C. jejuni* FliS and FliW protein toward the structural and secreted flagellins. Our results indicate that FliS and FliW are both required for bacterial motility but have different substrate specificity, require opposite ends of the structural FlaA flagellin for binding and display variable preference for binding to glycosylated FlaA/B flagellin. The results indicate the importance of *C. jejuni* FliS and FliW in flagella biogenesis and flagellin secretion.

## Materials and Methods

### Bacterial Strains, Growth Conditions, and Plasmids

The bacterial strains and plasmids used in the study are listed in Supplementary Materials and Methods (Supplementary Table S1). *Escherichia coli* strains were grown at 37°C in Luria Bertani (LB) broth or on LB plates (Biotrading). *Campylobacter* sp. were cultured on plates with 5% saponin-lysed horse blood (Biotrading) at 37°C, under microaerophilic conditions (5% O_2_, 7.5% CO_2_, 7.5% H_2_, 80% N_2_). When appropriate, growth media were supplemented with ampicillin (100 μg/ml) or chloramphenicol (20 μg/ml). Planktonic *Campylobacter* sp. cultures were grown in Heart Infusion (HI) broth (Biotrading) at 42°C, under microaerophilic conditions. Under these conditions, *C. jejuni* expresses predominantly FlaA flagellin as the *C. jejuni flaA* gene is highly expressed at 42°C, whereas the expression of *flaB* gene at this temperature is minimal (Wösten et al., 2010). This implies that FlaA flagellin is the main constituent of the flagellar filament at 42°C, as is also evident from the severely truncated or full-length filaments formed by the Δ*flaA* or Δ*flaB* knockout strain, respectively (Alm et al., 1993).

### Construction of the *C. jejuni* Δ*FliS* Strain

The primers used in the study are listed in Supplementary Materials and Methods (Supplementary Table S2).

The *fliS* gene (C8J_0510) and 1 kb of its flanking regions were amplified by PCR from the *C. jejuni* 81116 genome using the KR66–KR67 primer pair and cloned into the pJet1.2 vector. The resulting vector pJet1.2-*fliS* was used as a template in an outward PCR with the primer pair KR68–KR69. The PCR product was ligated to the *cat* cassette excised from the pAV35 vector with BamHI, yielding pJet1.2-*fliS*::*cat*. The *cat* cassette was inserted in the same orientation as the *fliS* ORF and replaced the central region of *fliS* gene leaving 50 and 100 nt at the 5′- and 3′-end of the *fliS* ORF, respectively (Supplementary Figure S1).

### Motility Assay

Bacterial swarming was assessed in 0.4% agar HI plates. One microliter of *C. jejuni* culture, diluted to OD_600_ of 1, was stabbed with a pipette into the plate. Motility zones were photographed after incubation at 42°C for 18 h under microaerophilic conditions. The experiment was repeated three times.

### Electron Microscopy

Mid-exponentially grown bacteria (OD_600_ 0.4–0.7) were fixed with 4% glutaraldehyde, 5 mM CaCl_2_, 10 mM MgCl_2_, in 0.1 M Na-cacodylate buffer. Samples were adhered to carbon-coated grids and stained with 0.5% uranylacetate. Imaging was done using a FEI Tecnai 12 electron microscope at 80 kV. Representative pictures were taken after observation of more than 50 bacteria.

### SDS-PAGE and Western Blot

Serially diluted *C. jejuni* lysates or supernatants were subjected to SDS-PAGE to analyze flagellin levels. Mid-exponentially grown *C. jejuni* cultures (OD_600_ 0.4–0.7) were adjusted to OD_600_ of 0.5. One ml of each culture was centrifuged (2,500 × *g*, 10 min) and supernatants were collected for further analysis. The supernatants were dialyzed against TBS buffer (20 mM Tris, 150 mM NaCl, pH 7.4) with two buffer changes. The initial dialysis, which was carried out at RT for 4 h with stirring, was followed by overnight dialysis at 4°C. Bacterial pellets were washed once with 1 ml of TBS and suspended in 1 ml of TBS. Bacteria were disrupted by sonication (Branson Sonifier, duty cycle 50%, output control 2, 30 s). The protein concentration was determined with the Pierce BCA Protein Assay Kit (Thermo Fisher Scientific). Protein samples (1,000, 500, 250, 125, and 62.5 ng of each sample) were separated by SDS-PAGE and transferred onto nitrocellulose using the Trans-Blot Turbo Transfer System (Bio-Rad). After incubation (1 h) of the blot with 5% skim milk in TBS-T (20 mM Tris, 150 mM NaCl, 0.05% Tween 20, pH 7.4), the membrane was probed (1 h) with primary antibodies diluted in 2% skim milk in TBS-T: polyclonal anti-FlaA/B (Nuijten et al., 1989) or anti-FlaC serum (Wösten et al., 2010), both diluted 1:10,000. After three 5-min washes with TBS-T, antibody binding was detected using goat anti-rabbit IgG conjugated with horseradish peroxidase (HRP; Sigma) diluted 1:10,000 in 2% skim milk in TBS-T. Reactive FlaA/B bands were visualized using SuperSignal West Pico Chemiluminescent Substrate (Thermo Fisher Scientific), whereas the detection of FlaC protein was carried out with SuperSignal West Femto Chemiluminescent Substrate (Thermo Fisher Scientific). Images were taken with ChemiDoc MP system (Bio-Rad). In all cases, at least three biological replicates were analyzed. To verify the equal protein loading, the samples were subjected to SDS-PAGE and stained by PageBlue Protein Staining Solution (Thermo Fisher Scientific) (Supplementary Figure S2).

His-tagged proteins were visualized with anti-His-HRP conjugate (Invitrogen). Glutathione *S*-transferase (GST)-tagged proteins were detected with anti-GST antibodies (Sigma, dilution: 1:10,000) and anti-mouse IgG antibody conjugated with HRP (Sigma, dilution 1:8,000). Reactive bands were visualized using SuperSignal West Pico Chemiluminescent Substrate (Thermo Fisher Scientific).

### Cloning of Recombinant Proteins

The pSCODON1.2 expression vector was used to construct FliS and RacR fused to a C-terminal His-tag. The *fliS* gene was amplified from pJet1.2-*fliS* with primers KR58 and KR59. The resulting PCR fragment was cloned directly into pSCODON1.2 with the use of NdeI and XhoI restriction enzymes, yielding pSCODON1.2-FliS. The cloning of *C. jejuni racR* gene to pSCODON1.2 vector has been described elsewhere (Radomska et al., 2016).

The pGEX4T-2 expression vector was used to create the N-terminal fusion of FliS with the GST tag. The *fliS* gene was amplified with the primer pair KR98-KR99 and the pJet1.2-*fliS* vector as a template. PCR product was directly cloned into pGEX4T-2, generating pGEX4T-2-FliS. EcoRI and XhoI sites present in the MCS of the vector were used for cloning. The cloning of *fliW* gene to pGEX4T-2 expression vector has been described elsewhere (Radomska et al., 2016).

The Champion pET101 Directional TOPO Expression Kit (Thermo Fisher Scientific) was used to create recombinant FlaA, FlaB, FlaC, and FliC flagellin in C-terminal fusion with the V5 epitope and the His-tag. Cloning was performed according to manufacturer’s instructions. The cloning of *C. jejuni flaA, flaB*, and *flaC* genes to pET101 vector has been previously described (Radomska et al., 2016). The predicted molecular weight of the tagged FlaA and FlaB flagellins is 63 kDa. The molecular weight of wild type (non-tagged) FlaA/B flagellin is 60 kDa but may increase (up to 10%) dependent on the degree of glycosylation (Thibault et al., 2001). The recombinant tagged FlaC flagellin has a predicted molecular weight of 29 kDa, whereas the native protein is 26 kDa in mass. The *fliC* gene of *S. enterica* sv. Enteritidis was amplified with FliC_hisF and FliC_hisR primers using the pT7.7-FliC vector (Keestra et al., 2008) as a template. The molecular weight of the tagged-FliC protein is 63 kDa.

To create FlaA protein fragments, the appropriate parts of the *flaA* gene were amplified using pET101-FlaA as a template. The following primer pairs were used: (1) FlaAΔC10: KR102-KR103, (2) FlaAΔC16: KR102-KR104, (3) FlaAΔC39: KR102-KR105, (4) FlaAΔND0: KR113-KR115, (5) FlaAΔCD0: KR102-KR116, (6) FlaAΔND1: KR114-KR115, (7) FlaAΔCD1: KR102-KR117. All PCR fragments were digested with NcoI and SacI restriction enzymes and ligated into the pET101 vector.

### Expression and Purification of Recombinant Proteins

The expression vectors were transformed to *E. coli* BL21 Star (DE3). Pre-cultures (16 h, 37°C) were used to inoculate 50 ml of LB broth (1:50). Bacteria were grown at 32°C until OD_600_ 0.5. Heterologous protein expression was induced by the addition of isopropyl-β-D-thiogalactopyranoside to a final concentration of 1 mM. After 4 h of induction bacteria were collected by centrifugation (4,000 × *g*, 15 min) and stored at -80°C.

His-tagged full length FlaA and the designed FlaA fragments were purified under denaturing conditions. Bacterial pellets were suspended in TBS buffer (pH 7.4) supplemented with EDTA-free complete protease inhibitor cocktail (Roche Diagnostics) and 10 mg/ml lysozyme (Sigma) and incubated on ice for 1 h. Bacteria were disrupted by sonication (10x 15 s pulses with 15 s holds on ice). The lysate was centrifuged (4,400 × *g*, 30 min, 4°C) and the soluble fraction was discarded. The insoluble pellet was suspended in 8 M urea, 20 mM Tris, 250 mM NaCl, 20 mM imidazole (pH 7.4) and incubated overnight at RT with end-over-end rotation. The next day 1 ml of Ni^2+^-NTA agarose beads (Thermo Fisher Scientific) was added and the incubation continued for 2 h. The mixture was loaded onto a column and the flow-through fraction was collected. The column was washed with 50 ml of 8 M urea, 20 mM Tris, 250 mM NaCl, 20 mM imidazole (pH 7.4). The His-tagged proteins were eluted with the 3 ml of 8 M urea, 20 mM Tris, 250 mM NaCl, 250 mM imidazole (pH 7.4). The proteins were dialyzed against TBS buffer (pH 9.0) with two buffer changes. The initial dialysis, which was carried out at RT for 4 h with stirring, was followed by overnight dialysis at 4°C. Recombinant flagellins were stored at -20°C in 4 M urea, 10 mM Tris (pH 9.0).

Glutathione *S*-transferase-tagged FliS, GST-tagged FliW and the non-fused GST-tag were purified under native conditions using Pierce Glutathione Agarose (Thermo Fisher Scientific) according to the manufacturer’s instructions. Briefly, bacteria were suspended in TBS buffer (pH 8.0) and disrupted by means of lysozyme and sonication as described above. Bacterial lysate was centrifuged (4,400 × *g*, 30 min, 4°C) and the supernatant representing the soluble fraction was mixed with 1 ml of Glutathione Agarose beads. The mixture was incubated 2 h at 4°C with end-over-end rotation and loaded on a column. After the flow-through was collected, the beads were washed with 100 ml of TBS (pH 8.0). Proteins were eluted with 3 ml of TBS (pH 8.0), containing 10 mM reduced glutathione. Eluted fractions were dialyzed overnight at 4°C against TBS (pH 8.0).

The purified proteins were dialyzed using SnakeSkin Dialysis Tubing, 10K MWCO (Thermo Fisher Scientific). The concentration of proteins was determined with Pierce BCA Protein Assay Kit (Thermo Fisher Scientific).

### Far Western Blot

To analyze protein–protein interaction by Far Western blot (affinity immunoblotting) 500 ng of total cell lysates of induced *E. coli* BL21 Star (DE3) harboring the appropriate expression vectors or 500 ng of purified proteins were separated by SDS-PAGE and transferred onto a nitrocellulose membrane. To analyze the cross-reactivity of *C. jejuni* FliS and FliW with flagellins from various *Campylobacter* sp. bacteria were grown overnight (stationary phase). The OD_600_ of the cultures was adjusted to 1 and 10 μl of each culture was loaded on SDS-PAGE gel. The membrane was probed with 50 μg of GST-FliS, GST-FliW or GST, followed by incubation with anti-GST antibodies (Sigma) diluted 1:10,000 and anti-mouse IgG antibody conjugated with HRP (Sigma), diluted 1:8,000. Fifty μg of each probe was used (**Figures 2, 5**–**7**), unless indicated otherwise (**Figure 4**). All probes were diluted in 2% skim milk TBS-T. Reactive bands were visualized using SuperSignal West Pico Chemiluminescent Substrate (Thermo Fisher Scientific).

To compare recognition of non-glycosylated and glycosylated flagellins by FliS, serial dilutions (500, 250, 125, and 62.5 ng) of His-tagged FlaA and the culture supernatant of strain BC7 that contains secreted *C. jejuni* flagellins were subjected to SDS-PAGE, blotted, and probed with 50, 100, 200 μg of GST-FliS or 200 μg of GST (**Figure 4**). In each case, at least two replicates were analyzed. Reactive bands were visualized using SuperSignal West Pico Chemiluminescent Substrate (Thermo Fisher Scientific).

### Pull-Down Assay

Pull-down experiments with His-tagged protein as a bait were performed as described previously (Radomska et al., 2016). Briefly, His-tagged FliS was solubilized with 8 M urea, 20 mM Tris, 250 mM NaCl, 20 mM imidazole (pH 7.4) and immobilized on Ni^2+^-NTA agarose beads. The protein was refolded on the beads with a set of buffers with the decreasing urea concentration: 8, 4, 2, 1, 0.5, 0.25, 0.13, 0.06 M urea solution in 20 mM Tris, 250 mM NaCl, 20 mM imidazole (pH 7.4); 5 ml of each buffer was used (40 ml in total). The final washing step was performed with 5 ml of 20 mM Tris, 250 mM NaCl, 20 mM imidazole (pH 7.4). The supernatant of a *C. jejuni* BC7 culture (grown at 42°C in 5 ml HI) was added to the nickel beads with immobilized FliS. The column was closed and incubated with end-over-end rotation for 1 h at RT. After washing (50 ml of 20 mM Tris, 250 mM NaCl, 20 mM imidazole, pH 7.4), His-tagged FliS together with the interacting partners was eluted with 1 ml of 20 mM Tris, 250 mM NaCl, 300 mM imidazole (pH 7.4). Samples collected during the procedure were analyzed by SDS-PAGE with PageBlue Protein Staining (Thermo Fisher Scientific) or by Western blotting using anti-His-HRP (Invitrogen), anti-FlaA/B (Nuijten et al., 1989), or anti-FlaC serum (Wösten et al., 2010). Control experiment was performed in analogous way, using RacR-His as a bait protein, which was refolded on the beads.

The GST-tagged proteins used as probes in Far Western blotting were also used as a bait in a pull-down assay (Supplementary Figure S3).

## Results

### Effect of Disruption of *C. jejuni FliS* on Bacterial Motility and Flagella Biosynthesis

Bioinformatic interrogation of the genome of *C. jejuni* strain 81116 indicated the gene C8J_0510 as the putative *fliS* homolog consistent with the annotation in the database. The gene was located in an operon together with the *flaG* gene encoding the putative flagellar protein FlaG, and the *fliD* gene that encodes the flagellar hook-associated protein FliD (Carrillo et al., 2004). The identified *C. jejuni* FliS protein sequence was highly conserved among *C. jejuni* strains (>94% identity) and was 60% identical at the amino acid level to the FliS of *H. pylori*, 32% to *S. enterica* FliS, and 28% to *A. aeolicus* FliS, respectively.

In order to define the putative role of the *C. jejuni* FliS protein in bacterial motility, we first disrupted the gene in *C. jejuni* strain 81116 by insertion of a *cat* cassette in the same orientation as *fliS* (Supplementary Figure S1A). The disrupted gene was introduced into strain 81116 by natural transformation (Wang and Taylor, 1990). Transcript analysis on RNA isolated from *C. jejuni* 81116 Δ*fliS* mutant demonstrated that insertion of the cassette did not disrupt the expression of the downstream gene encoding a hypothetical protein (Supplementary Figure S1B).

**FIGURE 1:**
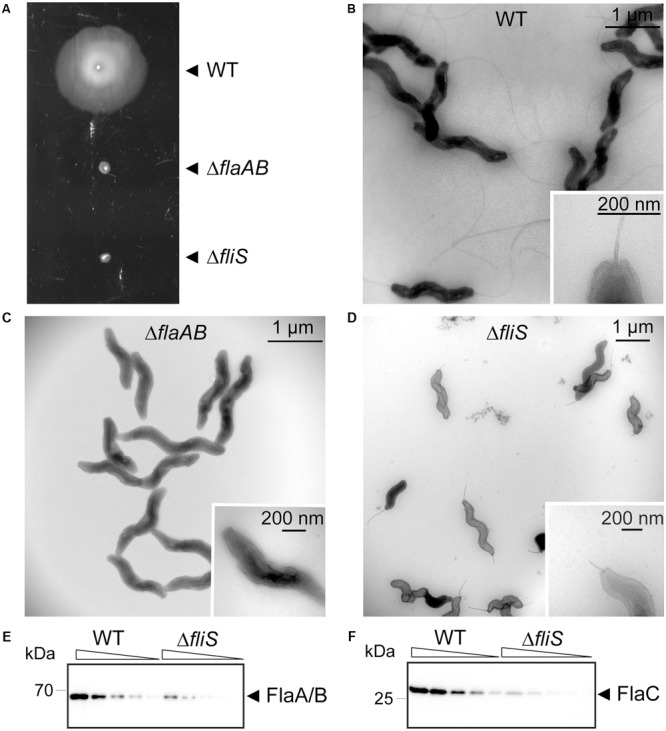
FliS is required for motility and flagellation of *Campylobacter jejuni*. **(A)** Motility of *C. jejuni* strain 81116 and its Δ*flaAB* and Δ*fliS* derivatives in HI broth supplemented with 0.4% agar after 18 h incubation at 42°C. **(B–D)** Transmission electron microscopy of the different *C. jejuni* strains showing the lack and severe truncation of flagella in the Δ*flaAB* and Δ*fliS* mutant strain. **(E,F)** Western blot demonstrating reduced flagellin levels in the Δ*fliS* mutant compared to the parent (WT) strain. Bacteria were grown in HI at 42°C till mid-exponential phase (OD_600_ 0.4–0.7). After adjustment of the OD_600_ to 0.5, serially diluted samples of bacterial lysates or supernatants were probed with polyclonal anti-FlaA/B or anti-FlaC serum, respectively. Molecular mass of proteins is indicated in kilodaltons (kDa).

To determine the effect of inactivation of *C. jejuni fliS* on bacterial motility and flagella biosynthesis, the phenotypes of *C. jejuni*Δ*fliS*, the wild type and Δ*flaAB* strain were compared. In the Δ*flaAB* mutant both adjacent flagellin-encoding genes are disrupted by insertion of the *cat* cassette (Wösten et al., 2004). Comparison of the Δ*fliS* strain, the parent strain, and a non-flagellated Δ*flaAB* mutant for their motility in soft agar demonstrated similar non-motile phenotypes for the Δ*fliS* and the Δ*flaAB* strain (**Figure 1A**). Transmission electron microscopy showed that the flagella in the Δ*fliS* mutant were severely truncated compared to the parent strain (**Figures 1B–D**), explaining the lack of motility of the mutant. In search for the basis of the lack of mature flagella in the Δ*fliS* mutant, the production of the FlaA and FlaB flagellins was verified by Western blotting. Hereto, serial dilutions of whole cell lysates of *C. jejuni* Δ*fliS* and the parent strain were probed with polyclonal antibodies recognizing both the FlaA and FlaB protein. The FlaA and FlaB flagellin are 95% identical and the molecular weight and antigenicity of the proteins are indistinguishable (Guerry et al., 1991; Nuijten et al., 1991). This showed that targeted disruption of the FliS-encoding gene strongly reduced the level of the FlaA/B flagellins (**Figure 1E**). We also compared the amounts of secreted FlaC flagellin in the culture supernatants of the mutant and wild type strain. This indicated that disruption of *fliS* also reduced the amount of FlaC in the medium (**Figure 1F**), suggesting a common effect of FliS on all three types of *C. jejuni* flagellin.

### Interaction of *FliS* with *C. jejuni* Flagellins

**FIGURE 2:**
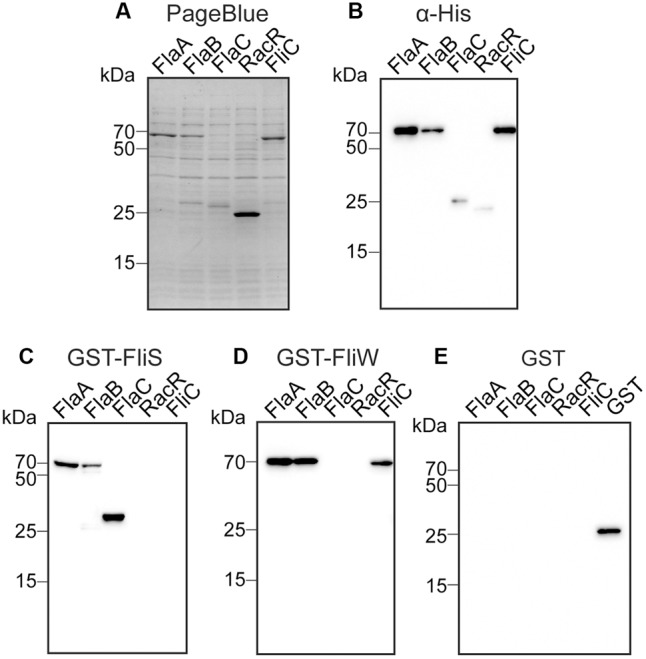
FliS interacts with recombinant FlaA, FlaB, and FlaC flagellin in Far Western blotting. Cell lysates of *Escherichia coli* expressing the indicated recombinant proteins were separated by SDS-PAGE and either **(A)** stained with PageBlue or **(B)** immunoblotted and probed with anti-His-HRP. The same protein samples were subjected to Far Western blotting, using 50 μg of purified **(C)** GST-FliS, **(D)** GST-FliW, or **(E)** GST as a probe. Binding was visualized using GST-specific antibodies and anti-mouse IgG antibody conjugated to HRP.

To investigate the ability of FliS to bind to the different flagellins, we analyzed the protein–protein interactions using Far Western blotting (affinity immunoblotting). The assay was performed with *E. coli* lysates expressing His-tagged FlaA, FlaB, or FlaC flagellin. The proteins were visualized by PageBlue staining or Western with anti-His-HRP conjugate (**Figures 2A,B**). The same protein samples were subjected to Far Western blotting, using purified GST-FliS, GST-FliW, or GST as probes (**Figures 2C–E**). As shown in **Figure 2C**, GST-FliS bound to all *C. jejuni* flagellins: FlaA, FlaB, and FlaC. No interaction was observed between *C. jejuni* FliS and the His-tagged FliC flagellin from *S. enterica* sv. Enteritidis or the motility-unrelated His-tagged RacR protein of *C. jejuni*, confirming the specificity of the interaction with the *C. jejuni* flagellins. The ability of FliS to bind all *C. jejuni* flagellins but not *Salmonella* FliC, differed from the substrate specificity of the *C. jejuni* flagellin binding protein FliW that senses and regulates flagella biosynthesis *via* a post-transcriptional mechanism (Radomska et al., 2016). The FliW protein of *C. jejuni* bound to the structural FlaA and FlaB flagellins and *S. enterica* FliC protein but not to *C. jejuni* FlaC (**Figure 2D**). None of the proteins was detected when the GST-tag was used as a probe in affinity immunoblotting (**Figure 2E**), confirming the specificity of observed interactions.

**FIGURE 3:**
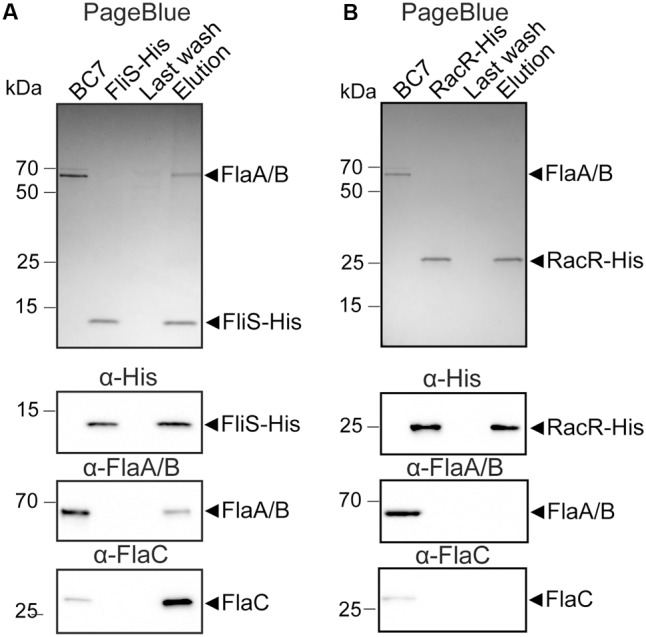
FliS interacts with all types of *C. jejuni* flagellins in pull down-assay. **(A)** SDS-PAGE and immunoblots of samples obtained before and after incubation of recombinant FliS protein (FliS-His) immobilized on nickel beads with culture supernatant of *C. jejuni* Δ*flgKM* (BC7) and during (Last wash) and after elution (Elution) of the bound proteins. Proteins were stained with PageBlue. The blots were probed with anti-His-HRP antibodies and anti-FlaA/B or anti-FlaC sera followed by anti-rabbit IgG conjugated with HRP. **(B)** Control pull-down assay performed in an analogous way but with the use of the RacR-His protein as a bait.

To assess the binding properties of FliS toward endogenous *C. jejuni* flagellins rather than the recombinant *E. coli* products, we immobilized His-tagged FliS on nickel agarose beads for use as a bait in pull-down experiments (**Figure 3A**). Culture supernatant of the *C. jejuni* strain BC7 which secretes high amounts of flagellins into the supernatant was used as a source of *C. jejuni* flagellins. In this strain the adjacent *flgK* and *flgM* genes are disrupted by the insertion of a kanamycin-resistance cassette (Bleumink-Pluym et al., 1999). The mutation of *flgK* gene, encoding a hook-filament junction protein, locks the synthesis of the flagellum at the assembly of the basal body and hook structure. The Δ*flgK* mutant is defective in formation of the hook filament junction, does not produce the filament, and secretes large amounts of flagellin into the medium (Barrero-Tobon and Hendrixson, 2014). The inactivation of *flgM* that encodes the anti-sigma factor, ensures the high level of expression of sigma^28^-dependent *flaA* gene (Wösten et al., 2010). Performed pull-down experiments showed that FliS was also able to capture *C. jejuni*-derived FlaA/B and FlaC flagellins, resulting in co-elution of FliS and the flagellins from the nickel column (**Figure 3A**). This result was not observed when the RacR-His protein rather than FliS-His was used as a bait (**Figure 3B**), confirming the specificity of observed interactions.

### Preferential Binding of *FliS* to Glycosylated *C. jejuni* Flagellins

**FIGURE 4:**
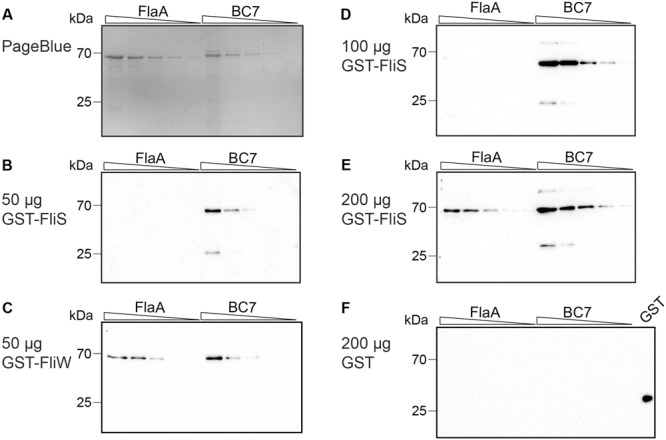
FliS recognizes preferentially glycosylated *C. jejuni* flagellins. Recombinant non-glycosylated *E. coli*-derived FlaA and the culture supernatant of BC7 strain containing secreted glycosylated *C. jejuni* flagellins were serially diluted and subjected to Far Western blotting. Proteins were visualized with **(A)** PageBlue staining or **(B–F)** transferred onto nitrocellulose. The membranes were probed with: **(B)** 50 μg of GST-FliS, **(C)** 50 μg of GST-FliW, **(D)** 100 μg of GST-FliS, **(E)** 200 μg of GST-FliS, or **(F)** 200 μg of GST (control). Binding of GST-tagged probes was detected using anti-GST mouse antibodies, followed by anti-mouse IgG-HRP. Note the preferential binding at the lower GST-FliS concentrations with the glycosylated *C. jejuni*-derived flagellins.

During *C. jejuni* flagella biosynthesis, the bacterial flagellins are synthesized and subsequently glycosylated (Ewing et al., 2009; Nothaft and Szymanski, 2010). The binding of FliS to recombinant and natural *C. jejuni* flagellins in the Far Western blot and pull-down assay respectively, indicates that FliS is capable to interact with the FlaA/B proteins independently of their glycosylation state. However, this does not exclude preferential binding of FliS to the non-glycosylated or glycosylated flagellins. To investigate whether the FliS protein displays preference for a distinct form of intracellular flagellin, we performed affinity immunoblotting on a series of concentrations of either *E. coli*-derived non-glycosylated FlaA or *C. jejuni* BC7 culture supernatant-derived glycosylated FlaA/B (**Figure 4**). Probing of the membranes with 50 μg of GST-FliS revealed that FliS displayed a clear preference for the *C. jejuni*-derived glycosylated FlaA/B and FlaC flagellins (**Figure 4B**). This preference was not observed when GST-FliW was used as a probe (**Figure 4C**). Additionally, we probed blots with 100 μg (**Figure 4D**) and 200 μg (**Figure 4E**) of GST-FliS. Again, FliS preferentially interacted with the glycosylated FlaA/B flagellin and with FlaC, while no binding was observed when 200 μg of GST was used as a probe (**Figure 4F**). These results clearly indicate that FliS favors binding to *C. jejuni*-derived flagellins.

Since recombinant FlaA flagellin is kept soluble in a solution containing 4 M urea, a control experiment was performed where urea powder was added to the *C. jejuni* BC7 culture supernatant to a final concentration of 4 M. Serial dilutions of His-tagged FlaA (in 4 M urea, 10 mM Tris, pH 9.0) and the culture supernatant of strain BC7 (with or without 4 M urea in TBS) were subjected to affinity immunoblotting (Supplementary Figure S4). The presence of urea in the sample of the *C. jejuni* BC7 supernatant did not alter interaction with GST-tagged FliS in Far Western blotting.

### Cross-Reactivity of *C. jejuni FliS* and FliW with Flagellins from Various *Campylobacter* sp.

**FIGURE 5:**
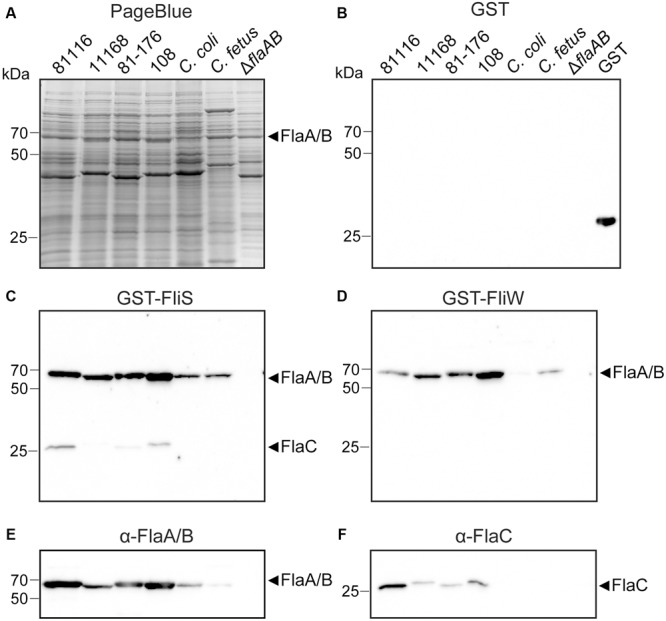
Flagellar proteins FliS and FliW bind to flagellins from various *Campylobacter* sp. Samples of liquid cultures of *C. jejuni* strains (81116, 11168, 81–176, 108), *C. coli* and *C. fetus* were separated by SDS-PAGE and stained with **(A)** PageBlue, or transferred onto nitrocellulose membrane and probed with **(B)** GST, **(C)** GST-FliS, or **(D)** GST-FliW. Binding of GST-tagged probes was detected with use of anti-GST mouse antibodies, followed by anti-mouse IgG conjugated with HRP. The presence of FlaA/B and FlaC flagellins in the samples was tested by Western blot using **(E)** polyclonal anti-FlaA/B or **(F)** anti-FlaC serum. Cell lysate of the *C. jejuni* Δ*flaAB* knockout strain served as a negative control.

To assess whether the diverse substrate specificity of *C. jejuni* FliS and FliW is a conserved trait among *Campylobacter* sp., we determined the binding of each of the proteins to flagellins from four *C. jejuni* strains (81116, 11168, 81–176, 108), *C. coli* and *C. fetus*. The *C. jejuni* Δ*flaAB* strain was used as a negative control. The presence of the flagellins in the bacterial cultures was confirmed by Western blot using FlaA/B- or FlaC-specific antisera. Probing of the blots with GST-FliS and GST-FliW revealed similar binding pattern as observed for the flagellin-specific antisera (**Figure 5**). FliS recognized the structural FlaA/B flagellin in all *Campylobacter* sp. tested. The FliW protein interacted with the FlaA/B flagellin of the different *C. jejuni* strains and, to a lesser extent, with the flagellin of *C. fetus*. Binding to the FlaA/B of *C. coli* was not observed. The secreted FlaC flagellin was detected by FliS in the majority of *C. jejuni* strains but not by FliW. Together, our data indicate that that the different substrate specificity of the FliS and FliW protein is a conserved trait among *Campylobacter* sp.

### Mapping of the *FliS* Binding Region in*C. jejuni* FlaA Flagellin

**FIGURE 6:**
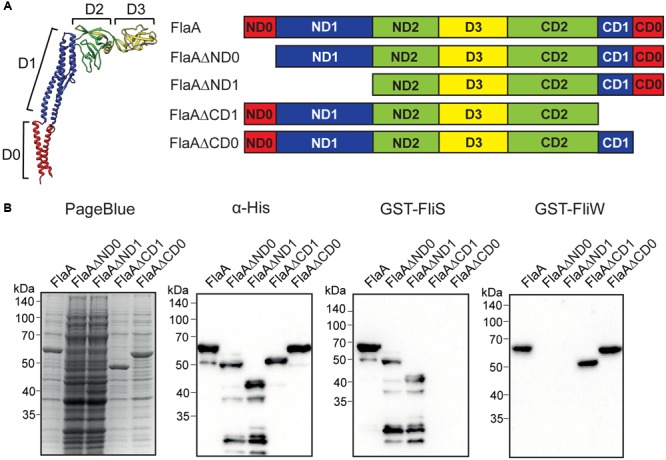
FliS and FliW interact with opposite subdomains in *C. jejuni* FlaA flagellin. **(A)** Flagellin domain organization presented on the structure of *Salmoneela enterica* FliC (PDB: 3A5X) and schematic representation of the constructed *C. jejuni* FlaA fragments used in the study. Flagellin domains are color-coded as following: red – D0, blue – D1, green – D2, yellow – D3. Each of the D0, D1, and D2 domain consists of N and C-terminal subdomains. **(B)** SDS-PAGE and immunoblots of lysates of *E. coli* expressing indicated recombinant flagellins. 500 ng of lysates of *E. coli* expressing FlaA, FlaAΔCD1, FlaAΔCD0 or 5 μg of *E. coli* expressing FlaAΔND0, FlaAΔND1 were separated by SDS-PAGE. Lysates containing FlaAΔND0 and FlaAΔND1 constructs were 10 times concentrated to ensure the detection of proteins. The proteins were visualized with PageBlue, the anti-His-HRP antibodies or the indicated GST-FliS or GST-FliW fusion proteins (50 μg), followed by anti-GST mouse antibodies and anti-mouse IgG-HRP.

As *C. jejuni* FliS binds to all three types of *C. jejuni* flagellins (FlaA, FlaB, and FlaC), we focused in search for the putative FliS binding region on the largely conserved D1 and D0 flagellin domains (Samatey et al., 2001; Song et al., 2004). Hereto, we produced a set of recombinant FlaA fragments. Truncated flagellins were cloned with a C-terminal His-tag, yielding *E. coli* expressing FlaAΔND0, FlaAΔND1, FlaAΔCD0, FlaAΔCD1 flagellins (**Figure 6** and Supplementary Figure S5). Protein staining and Western blotting using anti-His antibodies demonstrated strong protein expression of the fragments, except for both N-terminal truncated flagellins (FlaAΔND0 and FlaAΔND1) that were poorly expressed and rapidly degraded. For this reason, we tested in affinity immunoblotting the binding to 10x concentrated lysates of *E. coli* expressing FlaAΔND0 and FlaAΔND1 proteins (**Figure 6B**) as well as to unconcentrated samples (Supplementary Figure S5). Despite their poor expression, Far Western blotting using GST-FliS as a probe clearly showed binding of FliS to the FlaAΔND0 and FlaAΔND1 fragments but not to the strongly expressed C-terminally truncated flagellins (FlaAΔCD0 and FlaAΔCD1) (**Figure 6B** and Supplementary Figure S5). These findings indicate that CD0 subdomain of the *C. jejuni* FlaA flagellin is essential for FliS binding.

**FIGURE 7:**
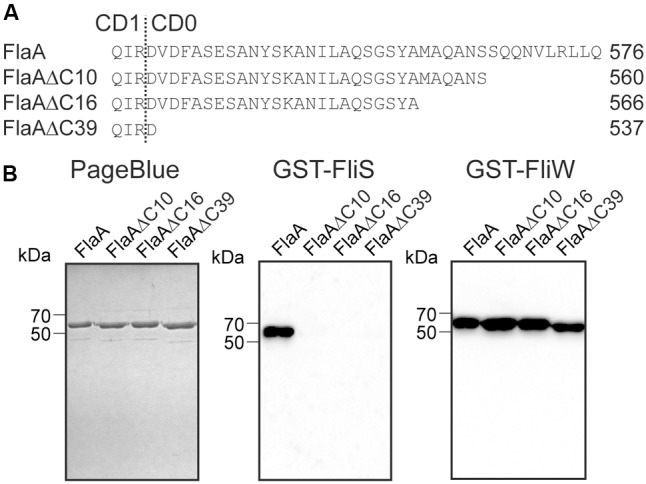
An intact C-terminus of FlaA flagellin is crucial for FliS recognition. **(A)** Carboxyterminal amino acid sequence of *C. jejuni* FlaA aligned with the truncated FlaAΔC10, FlaAΔC16, and FlaAΔC39 flagellins. The dotted line marks the transition between CD1 and CD0 subdomain. **(B)** SDS-PAGE and Far Western blot of full length *C. jejuni* FlaA and the truncated FlaAΔC10, FlaAΔC16, and FlaAΔC39 derivatives. Purified proteins (500 ng) were separated by SDS-PAGE and visualized by PageBlue staining or transferred onto a nitrocellulose membrane and probed with 50 μg of GST-FliS or GST-FliW. Binding of the probes was detected using anti-GST mouse antibodies and anti-mouse IgG-HRP.

As the CD0 subdomain of flagellins is highly conserved among *Campylobacter* sp. (Supplementary Figure S6 and Table S3) we attempted to further map the FliS binding site by constructing more defined truncations of the CD0 subdomain of the FlaA protein. This yielded the proteins FlaAΔC10, FlaAΔC16, and FlaAΔC39 that lacked respectively 10, 16, and 39 amino acids at their C-terminal ends (**Figure 7A**). Far Western blotting analysis using *E. coli* expressing these proteins and GST-FliS as a probe revealed that none of the truncated FlaA proteins was recognized by FliS (**Figure 7B**). This implies that an intact C-terminal end of *C. jejuni* flagellin is required for the binding of FliS.

### FliW Binding Region in *C. jejuni* FlaA Flagellin

In order to determine whether the flagellar binding proteins FliS and FliW compete for binding to flagellin due to overlapping bindings sites or bind to different regions of the *C. jejuni* flagellins, we mapped the binding region of the *C. jejuni* FliW protein using the series of constructed flagellin fragments. Probing of *E. coli* lysates expressing FlaAΔND0, FlaAΔND1, FlaAΔCD0, FlaAΔCD1 flagellins with purified GST-FliW showed that FliW recognized the FlaAΔCD0 and FlaAΔCD1 proteins but was unable to bind to the N-terminally truncated flagellins (FlaAΔND0 and FlaAΔND1) in contrast to FliS. Instead, FliW strongly reacted with the FlaAΔCD0 and FlaAΔCD1 flagellin fragments (**Figure 6B** and Supplementary Figure S5) and with the FlaAΔC10, FlaAΔC16, and FlaAΔC39 recombinant flagellins that lacked the FliS binding region (**Figure 7B**). These findings indicate that the N-terminal region of the flagellins is critical for the binding of *C. jejuni* FliW and thus that FliS and FliW intract with opposite ends of the protein.

## Discussion

**FIGURE 8:**
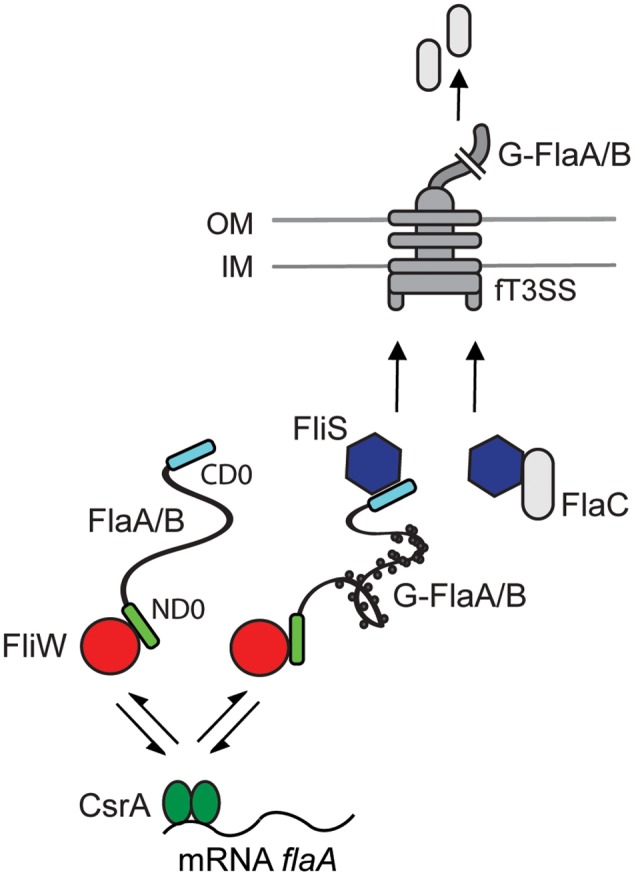
Proposed role of the flagellar proteins FliW and FliS in the intracellular processing of *C. jejuni* flagellins. Flagellar protein FliW (red circle) interacts with the ND0 subdomain of FlaA/B flagellin (green rectangle), but not with FlaC. FliW interacts with flagellin independently of its glycosylation state and acts as a sensor of cytoplasmic flagellin levels. FliW interacts reversibly (indicated by arrows) with the post-transcriptional regulator CsrA (Mukherjee et al., 2011; Dugar et al., 2016; Radomska et al., 2016). The FliS protein (blue hexagon) recognizes the CD0 subdomain of FlaA/B (blue rectangle) and preferentially binds to the glycosylated flagellin (G-FlaA/B). The preferential chaperone binding to the glycoform of flagellin ensures the selective secretion of glycosylated flagellin (Parker et al., 2014). FliW and FliS protein may interact with the glycosylated flagellin simultaneously (Mukherjee et al., 2013). FliS also interacts with FlaC, unlike FliW. Arrows indicate the targeting to the export gate and secretion of flagellins. Whereas G-FlaA/B flagellin is incorporated into the filament, FlaC is secreted to the environment. OM, outer membrane; IM, inner membrane; fT3SS, flagellar type 3 secretion system. Dots indicate glycan residues.

During bacterial flagella biosynthesis a set of specific molecular chaperones aids the proper export and assembly of flagella constituents. Most flagellar chaperones do not support protein folding but instead act as holding chaperones that prevent premature folding and polymerization of their partner proteins (Altegoer and Bange, 2015). Typically, the *E. coli* and *S. enterica* FlgN, FliS, and FliT chaperones prevent self-polymerization of the flagellar FlgK/FlgL, FliC, and FliD proteins, respectively (Fraser et al., 1999; Auvray et al., 2001; Khanra et al., 2016). Here, we provide evidence that the FliS ortholog of the major food-borne pathogen *C. jejuni* is essential for flagella assembly and bacterial motility. *C. jejuni* FliS binds to the filament components FlaA and FlaB as well as to the secreted FlaC protein. FliS prefers binding to the glycosylated flagellins, although its interaction domain is located in the (non-glycosylated) C-terminal end of the flagellin. The binding properties of FliS differ both with regard to substrate specificity and flagellin binding domain from the *C. jejuni* FliW protein (**Figure 8**). We show that this flagellar protein, which controls flagellin translation *via* a CsrA-dependent post-transcriptional regulatory mechanism (Dugar et al., 2016; Radomska et al., 2016), interacts with the N-terminal domain of FlaA and FlaB (but not FlaC) and has no preference for glycosylated flagellins. Preferential binding of FliS to glycosylated flagellins has also been reported for *Aeromonas caviae*. In this species the preferential chaperone binding to the glycoform of flagellin ensures the selective targeting to the export gate and secretion of glycosylated FlaA (Parker et al., 2014). When FliS and FliW are able to bind to the flagellin simultaneously, as reported for *B. subtilis* (Mukherjee et al., 2013), the proteins are likely recycled after delivery of the flagellin to the export gate. The mechanism behind this event remains to be determined. Together, our study demonstrates that FliS and FliW have different substrate specificity, interact with opposite ends of the structural FlaA flagellin, and display variable preference for binding to glycosylated FlaA/B flagellin (**Figure 8**).

The important role of FliS of *C. jejuni* 81116 in flagella filament formation and bacterial motility became directly evident from the presence of severely truncated flagella (**Figures 1A–D**) and the reduced levels of the structural FlaA/B flagellins (**Figure 1E**) in the constructed *C. jejuni* Δ*fliS* strain. This mutant phenotype resembles observations in *S. enterica, Pseudomonas aeruginosa, B. subtilis*, and *C. jejuni* strain 81–176 (Yokoseki et al., 1995; Capdevila et al., 2004; Mukherjee et al., 2011; Barrero-Tobon and Hendrixson, 2014). In *C. jejuni* 11168H inactivation of *fliS* results in defective biofilm formation (Joshua et al., 2006). We show that *C. jejuni* FliS is also vital for the secretion of the non-structural FlaC flagellin that is not incorporated into the flagella fiber but is secreted into the culture supernatant (**Figure 1F**). As the FlaC protein may play a role in the bacterial invasion of eukaryotic cells (Song et al., 2004), FliS may also be critical in this process. This is however difficult to verify as *C. jejuni* invasion requires FliS-dependent bacterial motility. The finding that *C. jejuni* FliS binds to all *C. jejuni* flagellins underpins the conclusions of a global *C. jejuni* yeast two-hybrid protein interaction screen (Parrish et al., 2007).

The binding of *C. jejuni* FliS to the structural flagellins FlaA and FlaB but also to the FlaC protein of different *Campylobacter* sp. can be explained by the relatively conserved ND0 and CD0 subdomains of these proteins (Supplementary Figure S6). This finding, in combination with the pattern of binding of FliS to constructed flagellin fragments, enabled us to indicate the C-terminal D0 subdomain of the flagellins as a critical binding site of *C. jejuni* FliS. More specifically, deletion of the last 10 amino acids of the protein was sufficient to prevent the binding of FliS. Within this region, the last three C-terminal amino acid residues (LLQ) of the FlaA, FlaB, and FlaC flagellins are most widely conserved among *Campylobacter* species (Supplementary Figure S6). The C-terminal D0 subdomain, which contributes to flagellin oligomerization, also acts as FliS binding region in distantly related enterobacterial species (Fraser et al., 1999; Auvray et al., 2001; Ozin et al., 2003). The interaction of this domain with FliS supposedly prevents premature intracellular folding of the flagellins and aids flagellin docking at the export gate of the flagellar T3SS (Kinoshita et al., 2013; Evans et al., 2014). In *H. pylori* FliS forms a complex with the co-chaperone molecule HP1076. The FliS-HP1076 complex was able to prevent DTT-induced insulin aggregation (Lam et al., 2010). As the amino acid sequence of FliS from *C. jejuni* and *H. pylori* show considerable identity (63.46%), we searched for the presence of a HP1076 homolog in the *C. jejuni* genome. As this revealed one candidate (C8J_1552) with rather low sequence identity (26%), this was not further investigated.

Recently, we and others identified the flagellar protein FliW of *C. jejuni* as a central element in the regulation of the amount of intracellular flagellin (Dugar et al., 2016; Radomska et al., 2016). FliW acts as an intracellular flagellin sensor and, through reciprocal binding to the post-transcriptional regulator CsrA, is part of a feedback loop that controls the translation of *flaA* and *flaB* transcripts. Here, we provide evidence that the FliW binding site is located in the N-terminal subdomain of *C. jejuni* FlaA. The binding of FliW to the N-terminal domain of *C. jejuni* flagellin is at variance with a previous study that reported interspecies recognition of flagellins by the FliW ortholog of *Treponema pallidum* and *B. subtilis* (Titz et al., 2006). In this study, a conserved asparagine (N) residue crucial for FliW binding was identified in the C-terminus of flagellins. The deletion of the N residue abolished FliW binding to Hag flagellin in *B. subtilis* (Titz et al., 2006). This conserved N residue is also present in all *C. jejuni* flagellins (Supplementary Figure S7). Yet, *C. jejuni* FliW interacts only with the FlaA and FlaB flagellin but not with the FlaC protein (**Figure 2**). This in conjunction with the consistent binding of *C. jejuni* FliW to the FlaAΔCD0 and FlaAΔCD1 flagellin fragments lacking the C-terminal D0 and D1 subdomains. That located the *C. jejuni* FliW binding site in the N-terminal domain of the flagellin. This means that *C. jejuni* FliW and FliS interact with opposite ends of FlaA/B flagellins.

An unexpected finding in our study was the apparent preferential binding of FliS to the glycosylated *C. jejuni* FlaA/B flagellins. The glycosylation of the structural flagellins increases their solubility and is considered to be a requirement for *C. jejuni* flagella assembly (Szymanski et al., 2003; Ewing et al., 2009). The attachment of sugars can increase the molecular weight of the flagellins by 10% (Thibault et al., 2001). The preferential binding of FliS to the glycosylated FlaA/B flagellins which as also been reported for the flagellin chaperone of *A. caviae* (Parker et al., 2014), suggests that flagellin glycosylation, which occurs in the central flagellin D2/D3 domains (Ewing et al., 2009; Ulasi et al., 2015), influences the ability of the C-terminal end of the protein to interact with FliS. How FliS senses the glycosylation status of the flagellin remains an open question. In a different biological setting, the glycosylated form of *P. aeruginosa* flagellin has been found to better activate TLR5 than its non-glycosylated counterpart. This suggests that also in this case glycosylation of the central D3 domain (Verma et al., 2005) influences the accessibility of distantly (in the D1 domain) located TLR5 binding site. More insight into the effects of glycosylation on the flagellin protein structure may explain these findings.

An intriguing finding in our study was the differential substrate specificity of *C. jejuni* FliW and FliS toward the secreted FlaC protein (**Figure 2**). One possible explanation for this observation may be that the intracellular level of the structural flagellins needs to be fine-tuned (by FliW) to accommodate optimal glycosylation and incorporation into the growing filament. In this scenario, binding of FliW to FlaC may not be required as this protein is not incorporated into the flagellum but directly secreted into the environment. Interestingly, FliS and FliW also differently interact with flagellin FliC of *S. enterica* (**Figure 2**). FliW was found to interact with *S. enterica* FliC, unlike FliS. It has been suggested that FliW-mediated CsrA regulation of flagellar motility is an ancestral regulatory mechanism of CsrA activity (Romeo et al., 2013; Altegoer et al., 2016). FliW and CsrA form an evolutionary coupled unit among flagellated bacteria, where FliW and the extended C-terminal end of CsrA that comprises the FliW-binding site, are subjected to co-evolution. The extended C-terminal end of CsrA was found to be present only in bacterial species encoding both CsrA and FliW, including *B. subtilis* and *C. jejuni*. The genomes of bacteria which CsrA activity is regulated by small RNAs such as *E. coli* and *S. enterica*, lack both the extended C-terminal end of CsrA and FliW (Altegoer et al., 2016). The observed interaction between FliW and *S. enterica* FliC flagellin may indicate a loss of *fliW* during the course of evolution of this species.

Bacterial flagellins are guided to the export gate using an N-terminal signal sequence (Supplementary Figure S7) reminiscent of the effectors molecules of related T3SS (Végh et al., 2006; Dobó et al., 2010). The structurally disordered region within the ND0 subdomain of *C. jejuni* flagellins, which is recognized by the flagellar export apparatus (Christensen et al., 2009; Neal-McKinney et al., 2010), seems to overlap with the FliW binding site. FliW has no preference of binding to glycosylated flagellins in contrast to FliS. On the basis of these data, it is tempting to speculate that when glycosylation of the flagellin is completed, FliW dissociates making the export signal available for targeting to the flagellar assembly machinery. A strong binding of FliS in this (FliW-free) state may be required to prevent premature folding of the glycosylated proteins. In this model, the binding of FliW may function to not only optimize intracellular flagellin levels (Dugar et al., 2016; Radomska et al., 2016) but also to prevent premature folding of the proteins. Thus, after glycosylation of the flagellins and dissociation of the flagellin sensor FliW, the FliS chaperone may primarily function to prevent pre-mature polymerization the glycosylated flagellins and to facilitate the flagellin delivery to the export gate. This model is supported by observations in *A. caviae*. In this species the flagellin chaperone preferentially binds to glycosylated flagellin resulting in preferred export of the glycoform of the protein (Parker et al., 2014). In *A. caviae* flagellin glycosylation occurs in the cytoplasm prior to chaperone binding. Since in *C. jejuni* flagellin glycosylation is essential for filament assembly (Goon et al., 2003; Logan, 2006), the preferential binding of FliS to glycosylated flagellins may serve to ensure the export of the glycosylated form needed for correct filament polymerization.

## Conclusion

We provided evidence that the *C. jejuni* flagellar proteins FliS and FliW interact with opposite ends of the structural flagellins FlaA and FlaB and differentially bind to the secreted FlaC protein. The FliS protein preferentially binds to the glycosylated flagellins and is vital for *C. jejuni* motility as well as the secretion of the FlaC protein, while the FliW protein mainly acts as sensor of intracellular FlaA/FlaB flagellin levels.

## Author Contributions

All authors listed, have made substantial, direct and intellectual contribution to the work, and approved it for publication. KR performed all the experiments, excluding TEM pictures which were taken by SO. KR and JvP wrote the manuscript.

## Conflict of Interest Statement

The authors declare that the research was conducted in the absence of any commercial or financial relationships that could be construed as a potential conflict of interest.
