# Supplementary material for: Importance of Campylobacter jejuni FliS and FliW in Flagella Biogenesis and Flagellin Secretion
Source: Front Microbiol. 2017 Jun 12;8:1060. doi: 10.3389/fmicb.2017.01060 (PMC5466977; doi:10.3389/fmicb.2017.01060)
Supplement: Supplementary file 1 [file Presentation_1.PDF]

Supplementary Materials

Importance of *Campylobacter jejuni* FliS and FliW in Flagella Biogenesis and Flagellin Secretion

Katarzyna A. Radomska[1], Marc M.S.M. Wösten[1], Soledad R. Ordoñez[1], Jaap A. Wagenaar[1,2,3], Jos P.M. van Putten[1,3*]

[1] Department of Infectious Diseases and Immunology, Utrecht University, Utrecht, the Netherlands

[2] Wageningen Bioveterinary Research, Lelystad, the Netherlands

[3] WHO Collaborating Centre for *Campylobacter*/ OIE Reference Laboratory for Campylobacteriosis

*Correspondence:
Jos P.M. van Putten
j.vanputten@uu.nl

Supplementary Figures

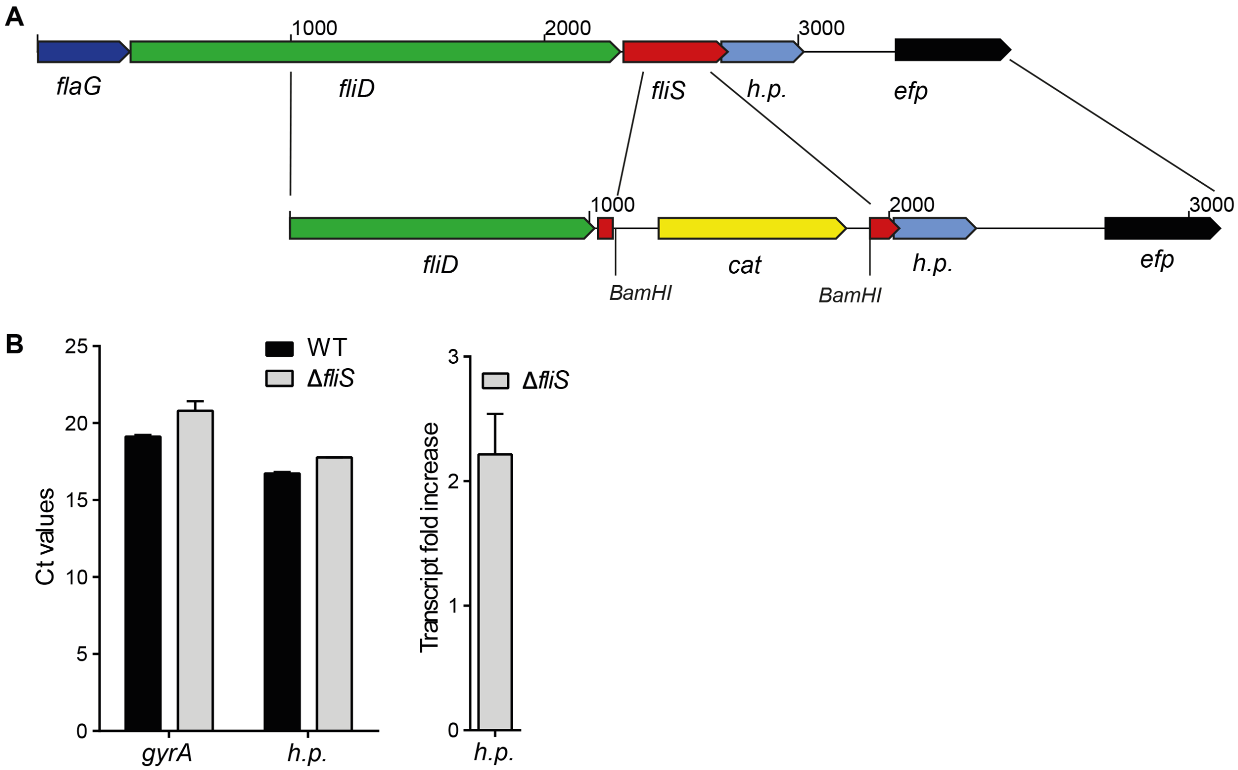

Figure S1. Construction strategy of the *C. jejuni* Δ*fliS* strain. (**A**) Physical map of the *fliS* gene locus (C8J_0510) in *C. jejuni* 81116 chromosome (upper panel) and schematic map of the deletion cassette used to construct *C. jejuni* Δ*fliS* strain (lower panel). The *cat* cassette (yellow) replaced the central part of *fliS* gene (red). Numbers above the map describe the size of the selected region in bp. Regions of homology between the *C. jejuni* chromosome and the deletion cassette are marked with lines. The following genes are depicted: *flaG* (C8J_0508) – the putative flagellar protein; *fliD* (C8J_0509) – the flagellar hook-associated protein; *h.p.* (C8J_0511) – the hypothetical protein; *efp* (C8J_0512) – the elongation factor P. *BamHI* was used for the insertion of the *cat* cassette. (**B**) Analysis of the polar effect of inactivation of *fliS* in *C. jejuni*. Transcript levels of the downstream *h.p.* gene (hypothetical protein, C8J_0511) and the *gyrA* reference gene were determined by real-time RT-PCR. The threshold cycle (Ct) for each gene amplification is displayed (left panel). Transcript levels in Δ*fliS* mutant are expressed as relative to the wild type strain (right panel).

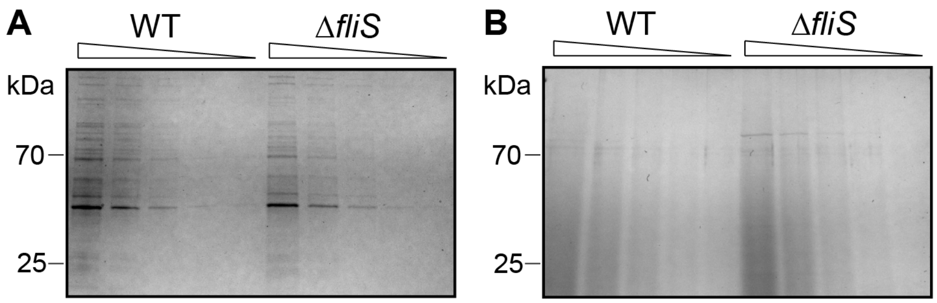

Figure S2. Analysis of flagellin levels in the *C. jejuni* wild type (WT) strain and Δ*fliS* mutant. Bacteria were grown in HI at 42°C until mid-exponential phase (OD_{600} 0.4-0.7). After adjustment of the OD_{600} to 0.5, serially diluted samples of (**A**) bacterial lysates or (**B**) supernatants were subjected to SDS-PAGE and visualized by PageBlue Protein Staining (Thermo Fisher Scientific).

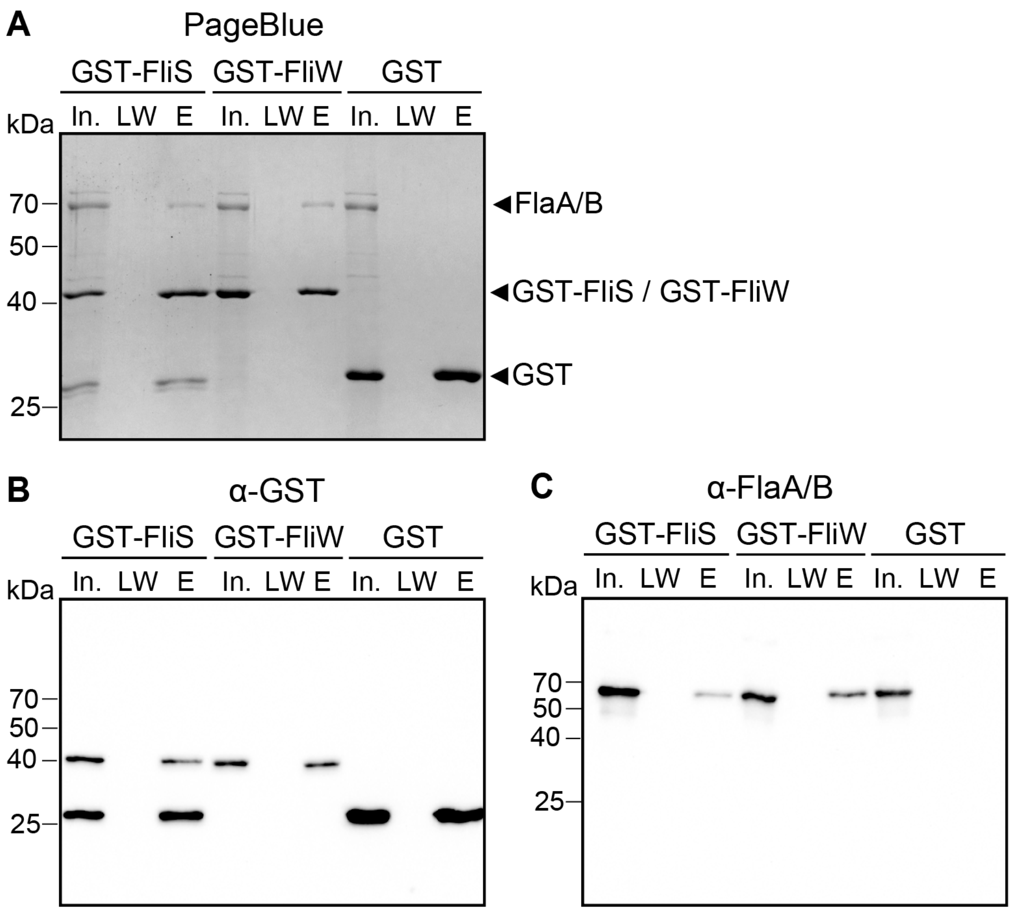

Figure S3. Pull-down assay with the GST-tagged proteins, which were used as a probe in Far Western blotting. The proteins: GST-FliS, GST-FliW or GST were pre-mixed with culture supernatant of *C. jejuni ΔflgKM* (BC7), which was used as a source of native flagellins. The protein mixtures were loaded onto glutathione agarose, washed extensively with TBS and eluted with the reduced L-glutathione in TBS. The samples collected during the procedure (In. – input, LW – last wash, E – elution) were (**A**) stained with PageBlue, or analyzed by Western blotting using (**B**) anti-GST antibodies (Sigma), (**C**) anti-FlaA/B (Nuijten et al., 1989), or anti-FlaC serum (Wösten et al., 2010). FlaC protein remained under the detection limit (data not shown). Note that the GST-FliS consists of full length GST-FliS (41 kDa) and shorter translation products. The mass of the N-terminal GST-tag is 26 kDa.

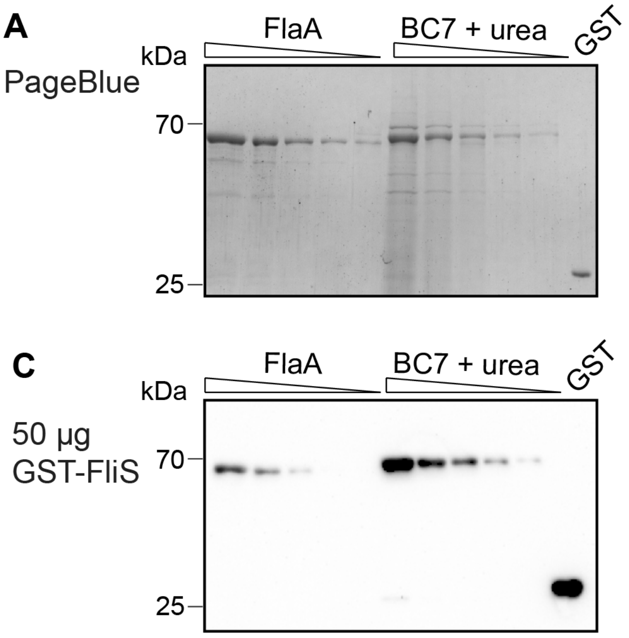

Figure S4. Urea does not influence the recognition of flagellins in Far Western blot. Urea powder was added to the *C. jejuni* BC7 culture supernatant to a final concentration of 4 M. Serial dilutions of the recombinant non-glycosylated *E. coli*-derived FlaA (in 4 M urea, 10 mM Tris pH 9.0) and the culture supernatant of strain BC7 (in TBS, with or without 4 M urea) were serially diluted and subjected to affinity immunoblotting. Proteins were visualized with (**A**) PageBlue staining or (**B**-**D**) transferred onto nitrocellulose and probed with (**B**) 50µg of GST or (**C**-**D**) 50µg of GST-FliS, followed by incubation with anti-GST mouse antibodies and anti-mouse IgG-HRP.

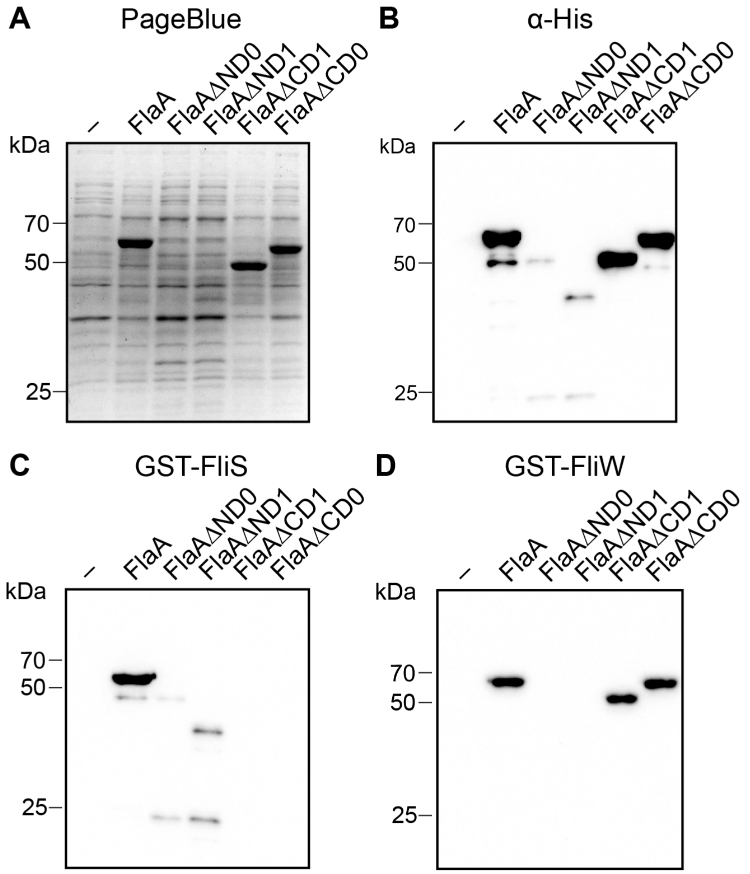

Figure S5. FliS and FliW bind to opposite subdomains of *C. jejuni* FlaA flagellin. Results of SDS-PAGE and immunoblots of total cell lysates of *E. coli* expressing FlaA, FlaAΔND0, FlaAΔND1, FlaAΔCD1, FlaAΔCD0 flagellins are depicted. Lysates (500 ng) of IPTG-induced *E. coli* BL21 Star (DE3) harboring the appropriate expression vectors or non-induced *E. coli* (negative control, indicated by a dash), were separated by SDS-PAGE and subjected to Western blotting. The proteins were visualized with: (**A**) PageBlue, (**B**) anti-His-HRP antibodies or (**C**) GST-FliS and (**D**) GST-FliW fusion proteins (50 µg), followed by anti-GST mouse antibodies and anti-mouse IgG-HRP.

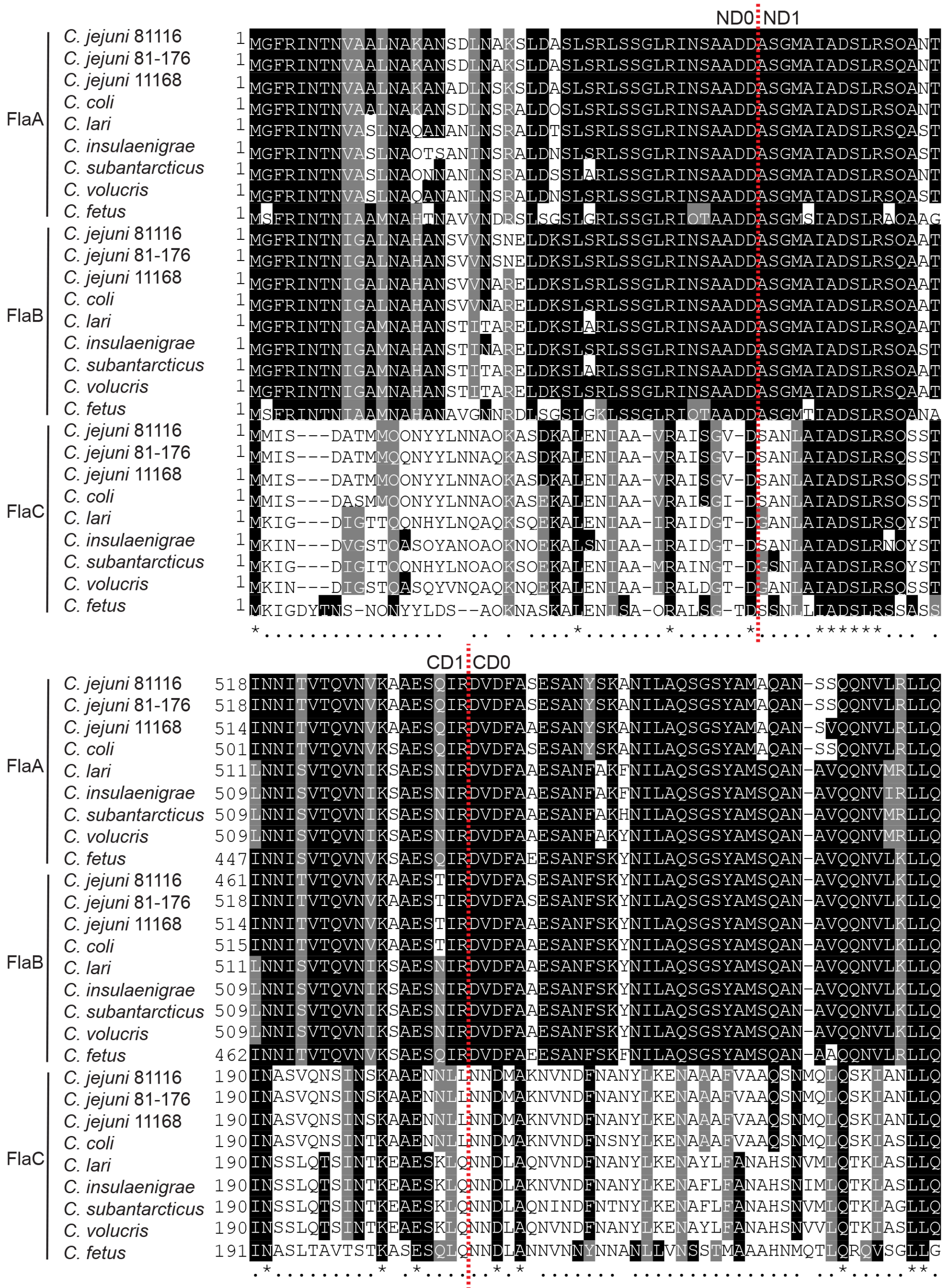

Figure S6. Sequence conservation of the N- and C-terminal ends of FlaA, FlaB and FlaC flagellins among various *Campylobacter* sp. Multiple sequence alignment was performed with T-Coffee. Amino acid sequence identifiers are listed in Table S3. Homologous regions are highlighted in black (identical amino acid residues) and grey (conserved amino acid substitutions). The consensus symbols indicate: 1) an asterisk – a fully conserved residue, 2) a period – amino acids of weakly similar properties. The red dotted lines mark the boundaries of the flagellin subdomains.

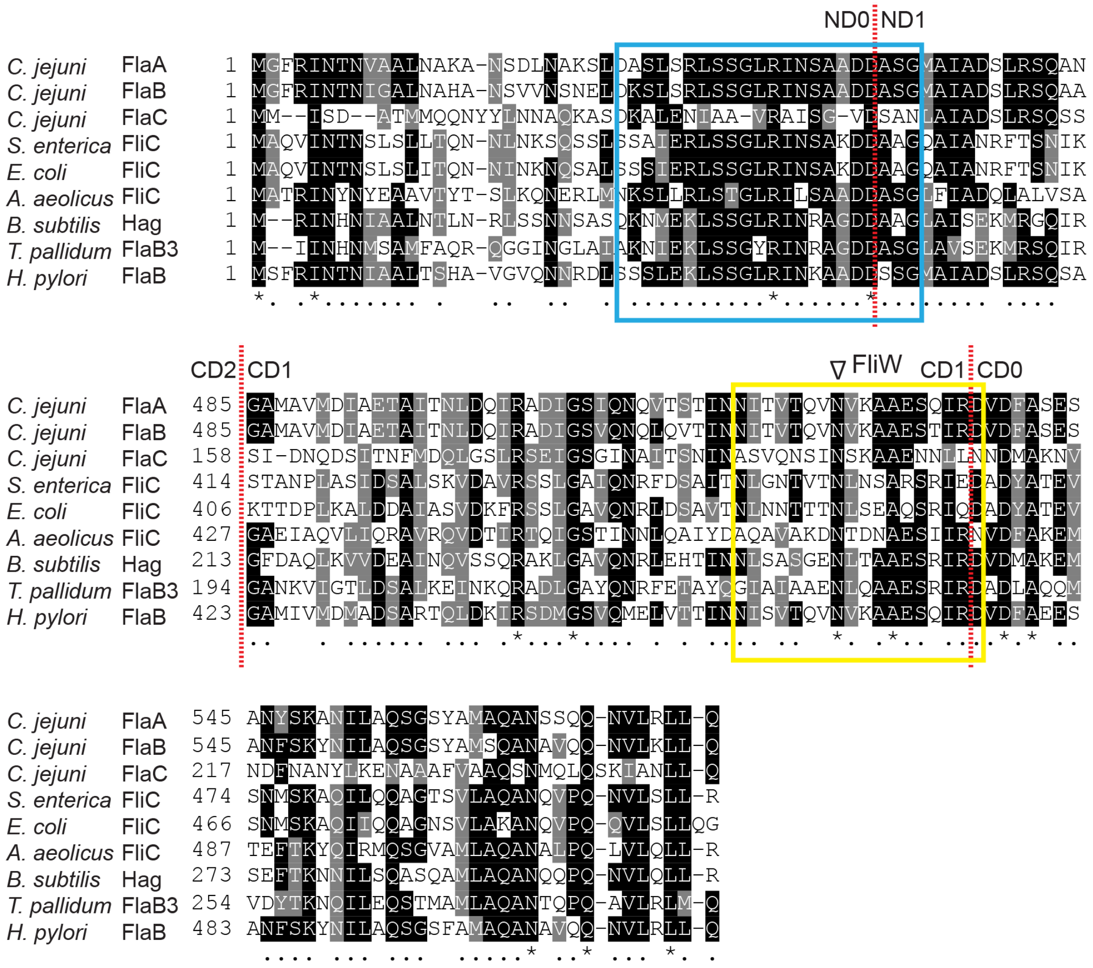

Figure S7. FliS and FliW binding sites within flagellins from different bacterial species. Multiple sequence alignment (T-Coffee) of amino acid sequences of the N- and C-terminal ends of flagellins from various bacterial species (sequences identifiers are listed in Table S3). The 22-residue long segment within the ND0 region of *Salmonella enterica* flagellin containing the recognition signal for the flagellar export system (Végh et al., 2006) is boxed in blue. The *Treponema pallidum* flagellin epitope responsible for interaction with FliW (Titz et al., 2006) is boxed in yellow. The conserved asparagine residue (N) which deletion abolished FliW binding to Hag flagellin in *Bacillus subtilis* is marked with triangle (Titz et al., 2006). Homologous regions are highlighted in black (identical amino acid residues) and grey (conserved amino acid substitutions). The consensus symbols indicate: 1) an asterisk – a fully conserved residue, 2) a period – amino acids of weakly similar properties. The boundaries between flagellin subdomains are marked with red dotted lines.

Supplementary Tables

Table S1. Bacterial strains and plasmids used in this study.

Strain/ Plasmid	Genotype/ Description*	Source/ Reference
***Campylobacter* sp.**		
C. jejuni 81116	Wild type (WT); human enteritis	(Palmer et al., 1983)
C. jejuni 11168	Wild type; human enteritis	(Skirrow, 1977)
C. jejuni 81-176	Wild type; human enteritis	(Black et al., 1988)
C. jejuni 108	Wild type; bacteremia	(Endtz et al., 1993)
C. coli	Wild type; human stool	(Wenman et al., 1985)
C. fetus	Wild type; human blood	(Perez et al., 1985)
C. jejuni Δ*fliS*	81116 *fliS*:: *cat*	This study
C. jejuni Δ*flaAB*	81116 *flaAB*:: *cat*	(Wösten et al., 2010)
C. jejuni Δ*flgKM*	81116 *flgKM*:: *aph* (BC7)	(Bleumink-Pluym et al., 1999)
***E. coli* strains**		
E. coli PC2955	*relA1,* Φ*80dlacZ* ΔM15, *phoA8, hsdR17, recA1 endA1, gyrA96, thi-1, luxS, glnV44*	NCCB
E. coli BL21 Star (DE3)	F^- *ompT hsdS*$_B$ (r_B^-, m_B^-) *gal dcm rne*131 (DE3)	Thermo Fisher Scientific
**Plasmids**		
pJet1.2/blunt	Cloning vector, Amp^R	Thermo Fisher Scientific
pAV35	pBluescript KS M13$^+$ ::Cm^R (*C. coli*)	(van Vliet et al., 1998)
pJet1.2-*fliS*	*fliS* gene with flanking regions cloned to pJet1.2	This study
pJet1.2-*fliS*:: *cat*	*fliS* gene disrupted with *cat* cassette (from pAV35)	This study
pSCODON1.2	Expression vector, His-tag (C-terminal fusion), Amp^R	Eurogentec
pSCODON1.2-FliS	*fliS* gene cloned to pSCODON1.2 vector	This study
pSCODON1.2-RacR	*racR* gene cloned to pSCODON1.2 vector	(Radomska et al., 2016)
pGEX4T-2	Expression vector, GST-tag (N-terminal fusion), Amp^R	GE Healthcare Life Sciences
pGEX4T-2-FliS	*fliS* gene cloned to pGEX4T-2 vector	This study
pGEX4T-2-FliW	*fliW* gene cloned to pGEX4T-2 vector	(Radomska et al., 2016)
pET101	Expression vector, His-tag (C-terminal fusion), Amp^R	Thermo Fisher Scientific
pET101-FlaA	*flaA* gene cloned to pET101 vector (full length FlaA: 1-576 aa)	(Radomska et al., 2016)
pET101-FlaB	*flaB* gene cloned to pET101 vector	(Radomska et al., 2016)
pET101-FlaC	*flaC* gene cloned to pET101 vector	(Radomska et al., 2016)
pET101-FliC	*fliC* gene cloned to pET101 vector	This study
pT7.7-FliC	*fliC* gene of *S. enterica* sv. Enteritidis cloned to pT7.7 vector	(Keestra et al., 2008)

pET101-FlaAΔC10	Fragment of *flaA* gene encoding FlaAΔC10 (1-565 aa of FlaA) cloned to pET101 vector	This study
pET101-FlaAΔC16	Fragment of *flaA* gene encoding FlaAΔC16 (1-559 aa of FlaA) cloned to pET101 vector	This study
pET101-FlaAΔC39	Fragment of *flaA* gene encoding FlaAΔC39 (1-537 aa of FlaA) cloned to pET101 vector	This study
pET101-FlaAΔND0	Fragment of *flaA* gene encoding FlaAΔND0 (45-576 aa of FlaA) cloned to pET101 vector	This study
pET101-FlaAΔND1	Fragment of *flaA* gene encoding FlaAΔND1 (177-576 aa of FlaA) cloned to pET101	This study
pET101-FlaAΔCD0	Fragment of *flaA* gene encoding FlaAΔCD0 (1-536 aa of FlaA) cloned to pET101 vector	This study
pET101-FlaAΔCD1	Fragment of *flaA* gene encoding FlaAΔCD1 (1-484 aa of FlaA) cloned to pET101 vector	This study

* aa – amino acids

Table S2. Primers used in this study.

Primer name	DNA sequence (5'-3')*
FliC_hisF	CACCATGGCACAAGTCATTAATACAAACAGCCTGTC
FliC_hisR	CGCAGTAAAGAGAGGACGTTTTG
KR102	TCACCATGGGATTTCGTATTAACAC
KR103	GTAGAGCTCAGAATTTGCTTGAGCCATTGC
KR104	GTAGAGCTCTGCATAAGAACCACTTTGGGC
KR105	GTAGAGCTCATCTCTGATTTGCGATTCTGC
KR113	TCACCATGGCTTCAGGGATGGCG
KR114	TCACCATGGGTGCTCAAAGTTTTAC
KR115	GTAGAGCTCGCCCTTTTGTAATAATC
KR116	GTAGAGCTCTCTGATTTGCGATTCTG
KR117	GTAGAGCTCTTTAAGAGTGGTTACAC
KR58	TGCCATATGCAAAATAATTTAGC
KR59	TACCTCGAG CTGAGCCACTGTTTC
KR66	AAAGCCATGCAAGATTTGGTG
KR67	GGAACTTCTACGCCGATAGC
KR68	GCTGGATCCATCCCCGCTTGATTTTGAGAATAAG
KR69	GACGGATCCAGAAAATAACGAAGATAGAATCAATGAAG
KR98	TGAGAATTCTT ATGCAAAATAATTTAGC
KR99	GTACTCGAGTCACTGAGCCACTGTTTC
MW670	ACGACTTACACGACCGATTTCA
MW671	ATGCTCTTTGCAGTAACCAAAAAA
RT1	ATCGCCTTCAGAAGATTTTTTAA
RT2	CAGCATAACAGCTTCTTGCAA

*Restriction sites used for cloning are underlined.

Table S3. Sequence identifiers (GenBank, NCBI) of flagellins used in multiple sequence alignments.

Species	Flagellin name	GenBank, NCBI
C. jejuni subsp. *jejuni* 81116	FlaA	ABV52855
C. jejuni subsp. *jejuni* 81116	FlaB	ABV52854
C. jejuni subsp. *jejuni* 81116	FlaC	ABV52286
C. jejuni subsp. *jejuni* 81-176	FlaA	EAQ72691
C. jejuni subsp. *jejuni* 81-176	FlaB	EAQ72883
C. jejuni subsp. *jejuni* 81-176	FlaC	EAQ72421
C. jejuni subsp. *jejuni* NCTC 11168	FlaA	YP_002344727
C. jejuni subsp. *jejuni* NCTC 11168	FlaB	YP_002344726
C. jejuni subsp. *jejuni* NCTC 11168	FlaC	YP_002344138
C. coli CVM N29710	FlaA	AGV09555
C. coli CVM N29710	FlaB	AGV09556
C. coli CVM N29710	FlaC	AGV10149
C. lari RM2100	FlaA	ACM63526
C. lari RM2100	FlaB	ACM63527
C. lari RM2100	FlaC	ACM63901
C. insulaenigrae	FlaA	AJC87155
C. insulaenigrae	FlaB	AJC87156
C. insulaenigrae	FlaC	AJC87476
C. subantarcticus LMG 24377	FlaA	AJC91737
C. subantarcticus LMG 24377	FlaB	AJC91738
C. subantarcticus LMG 24377	FlaC	AJC92124
C. volucris	FlaA	AJC93502
C. volucris	FlaB	AJC93503
C. volucris	FlaC	AJC93831
C. fetus subsp. *fetus* 04/554	FlaA	CFF04554_1637
C. fetus subsp. *fetus* 04/554	FlaB	CFF04554_1638
C. fetus subsp. *fetus* 04/554	FlaC	CFF04554_0512
S. enterica subsp. *enterica* sv. Enteritidis	FliC	EPI99153
E. coli	FliC	KDU30170
Aquifex aeolicus	FliC	NP_214372
Bacillus subtilis	Hag	BAM55613
Treponema pallidum	FlaB3	ACD71286
Helicobacter pylori	FlaB	AAA25016

Supplementary Materials and Methods

Real-time RT-PCR analysis

Real-time RT-PCR (RT-qPCR) analysis was performed as previously described (Radomska et al., 2016). Primers used in the assay (*gyrA*: MW670-MW671, *fliS*: NB62-NB63) are listed in Table S2. The calculated threshold cycle (Ct) for each gene amplification was normalized to the Ct value for *gyrA* gene, amplified of the corresponding sample, before calculating fold change using the arithmetic formula ($2^{-\Delta\Delta Ct}$), where ΔΔCt = [(Ct target gene - Ct *gyrA*) mutant - (Ct target gene - Ct *gyrA*) wild type] (Livak and Schmittgen, 2001). Each sample was examined in four replicates and was repeated with two independent preparations of RNA.

Pull-down assay

The GST-tagged proteins, which were used as a probe in Far Western blotting, were also used as a bait in a pull-down assay. Fifty µg of GST-FliS, GST-FliW or GST was mixed with 50 µl of the supernatant of a *C. jejuni* BC7 culture (grown at 42°C in 5 ml HI). The final volume of each mixture was adjusted to 400 µl with TBS (20 mM Tris, 250 mM NaCl, pH 8.0) and the control sample was collected (input). Subsequently, 12.5 µl of the Pierce Glutathione Agarose (Thermo Fisher Scientific) and 100 µl of 5% skim milk in TBS were added. Each mixture was incubated at RT for 1 h with end-over-end rotation. After incubation, each sample was centrifuged (1 min at 800 × g) and supernatant was discarded. The unbound proteins were washed away with 10 ml of TBS (10 cycles: 1 ml of TBS, centrifugation for 1 min at 800 × g). After the last washing cycle, 60 µl of TBS were added, the sample was centrifuged and 50 µl of the supernatant was collected as a control sample (last wash). GST-tagged proteins were eluted with 60 µl of 25 mM reduced L-glutathione (Sigma-Aldrich) in TBS. After centrifugation (1 min at 800 × g) 50 µl of the supernatant was collected (elution). The samples collected during the procedure (input, last wash, elution) were analyzed by SDS-PAGE with PageBlue Protein Staining (Thermo Fisher Scientific) or by Western blotting using anti-GST antibodies (Sigma), anti-FlaA/B (Nuijten et al., 1989), or anti-FlaC serum (Wösten et al., 2010).

Far Western blot

To analyze the influence of the urea on the recognition of FlaA/B flagellin present in the supernatant of *C. jejuni* BC7 strain, urea powder was added to the culture supernatant to a final concentration of 4 M. Serial dilutions (500 ng, 250 ng, 125 ng and 62.5 ng) of His-tagged FlaA (in 4 M urea, 10 mM Tris pH 9.0) and the culture supernatant of strain BC7 (in TBS, with or without 4 M urea) were subjected to SDS-PAGE, blotted, and probed with 50 µg of GST-FliS or 50 µg GST. Binding of the probes was detected with anti-GST antibodies (Sigma) diluted 1:10,000 and anti-mouse IgG antibody conjugated with HRP (Sigma), diluted 1:8,000. All probes and antibodies were diluted in 2% skim milk TBS-T. Reactive bands were visualized using SuperSignal West Pico Chemiluminescent Substrate (Thermo Fisher Scientific).

In silico analyzes

Multiple sequence alignments were performed with T-Coffee program (Notredame et al., 2000). Produced alignments were processed with BOXSHADE tool available at ExPASy

(http://embnet.vital-it.ch/software/BOX_form.html). Sequences used in the alignment are identified in Table S3.

Supplementary References

Black, R. E., Levine, M. M., Clements, M. L., Hughes, T. P., and Blaser, M. J. (1988). Experimental *Campylobacter jejuni* infection in humans. *J. Infect. Dis.* 157, 472–479.

Bleumink-Pluym, N. M. C., Verschoor, F., Gaastra, W., van der Zeijst, B. A. M., and Fry, B. N. (1999). A novel approach for the construction of a *Campylobacter* mutant library. *Microbiology* 145, 2145–2151. doi:10.1099/13500872-145-8-2145.

Endtz, H. P., Giesendorf, B. A., van Belkum, A., Lauwers, S. J., Jansen, W. H., and Quint, W. G. (1993). PCR-mediated DNA typing of *Campylobacter jejuni* isolated from patients with recurrent infections. *Res. Microbiol.* 144, 703–708.

Keestra, A. M., de Zoete, M. R., van Aubel, R. A. M. H., and van Putten, J. P. M. (2008). Functional characterization of chicken TLR5 reveals species-specific recognition of flagellin. *Mol. Immunol.* 45, 1298–1307. doi:10.1016/j.molimm.2007.09.013.

Livak, K. J., and Schmittgen, T. D. (2001). Analysis of relative gene expression data using real-time quantitative PCR and the 2−ΔΔCT method. *Methods* 25, 402–408. doi:10.1006/meth.2001.1262.

Notredame, C., Higgins, D. G., and Heringa, J. (2000). T-coffee: a novel method for fast and accurate multiple sequence alignment. *J. Mol. Biol.* 302, 205–217. doi:10.1006/jmbi.2000.4042.

Palmer, S. R., Gully, P. R., White, J. M., Pearson, A. D., Suckling, W. G., Jones, D. M., et al. (1983). Water-borne outbreak of campylobacter gastroenteritis. *Lancet Lond. Engl.* 1, 287–290.

Perez, G. I., Hopkins, J. A., and Blaser, M. J. (1985). Antigenic heterogeneity of lipopolysaccharides from *Campylobacter jejuni* and *Campylobacter fetus*. *Infect. Immun.* 48, 528–533.

Radomska, K. A., Ordoñez, S. R., Wösten, M. M. S. M., Wagenaar, J. A., and van Putten, J. P. M. (2016). Feedback control of *Campylobacter jejuni* flagellin levels through reciprocal binding of FliW to flagellin and the global regulator CsrA. *Mol. Microbiol.* doi:10.1111/mmi.13455.

Skirrow, M. B. (1977). *Campylobacter* enteritis: a “new” disease. *Br. Med. J.* 2, 9–11.

Titz, B., Rajagopala, S. V., Ester, C., Häuser, R., and Uetz, P. (2006). Novel conserved assembly factor of the bacterial flagellum. *J. Bacteriol.* 188, 7700–7706. doi:10.1128/JB.00820-06.

van Vliet, A. H. M., Wooldridge, K. G., and Ketley, J. M. (1998). Iron-responsive gene regulation in a *Campylobacter jejuni fur* mutant. *J. Bacteriol.* 180, 5291–5298.

Végh, B. M., Gál, P., Dobó, J., Závodszky, P., and Vonderviszt, F. (2006). Localization of the flagellum-specific secretion signal in *Salmonella* flagellin. *Biochem. Biophys. Res. Commun.* 345, 93–98. doi:10.1016/j.bbrc.2006.04.055.

Wenman, W. M., Chai, J., Louie, T. J., Goudreau, C., Lior, H., Newell, D. G., et al. (1985). Antigenic analysis of *Campylobacter* flagellar protein and other proteins. *J. Clin. Microbiol.* 21, 108–112.

Wösten, M. M. S. M., van Dijk, L., Veenendaal, A. K. J., de Zoete, M. R., Bleumink-Pluijm, N. M. C., and van Putten, J. P. M. (2010). Temperature-dependent FlgM/FliA complex formation regulates *Campylobacter jejuni* flagella length. *Mol. Microbiol.* 75, 1577–1591. doi:10.1111/j.1365-2958.2010.07079.x.
